# Synthesis and Characterization of a Novel Cassava Starch-Based Scaffold Biofunctionalized with Decellularized Extracellular Matrix and Isosorbide Dinitrate

**DOI:** 10.3390/polym17101307

**Published:** 2025-05-10

**Authors:** Samantha Dení Cabo-Araoz, Bernardino Isaac Cerda-Cristerna, Diana María Escobar-García, José Manuel Gutiérrez-Hernández, Mariana Gutiérrez-Sánchez, Amaury Pozos-Guillén, Héctor Flores

**Affiliations:** 1 Basic Science Laboratory, Faculty of Dentistry, University of San Luis Potosí, Av. Dr. Manuel Nava 2, San Luis Potosí 78290, SLP, Mexico; a363215@alumnos.uaslp.mx (S.D.C.-A.); diana.escobar@uaslp.mx (D.M.E.-G.); jose.manuel.gutierrez@uaslp.mx (J.M.G.-H.); heflores@uaslp.mx (H.F.); 2 Doctorado Institucional en Ingeniería y Ciencia de Materiales, Faculty of Dentistry, University of San Luis Potosí, Av. Dr. Manuel Nava 2, San Luis Potosí 78290, SLP, Mexico; 3 Faculty of Dentistry, Orizaba-Córdoba, University of Veracruz, Abasolo Sur S/N, Tenango de Río Blanco 94732, VE, Mexico; bcerda@uv.mx; 4 Endodontics Postgraduate Program, Faculty of Dentistry, University of San Luis Potosí, Av. Dr. Manuel Nava 2, San Luis Potosí 78290, SLP, Mexico; mariana.gutierrez@uaslp.mx

**Keywords:** biopolymeric system, starch, ECD, ISDN

## Abstract

This study aimed to synthesize and characterize cassava starch-based (S) scaffolds functionalized with decellularized extracellular matrix (dECM) and isosorbide dinitrate (ISDN) for wound healing. The scaffolds were synthesized via the casting method and evaluated for physicochemical, mechanical, and morphological properties, as well as ISDN release and hemocompatibility. Swelling and degradation tests revealed a biphasic behavior, with high water absorption followed by controlled degradation. The ISDN release followed a biphasic pattern, fitting the Korsmeyer–Peppas model. Hemolysis tests confirmed biocompatibility, with hemolysis levels below 2%. Among the formulations, the scaffold containing 12.5% ECM and 40 mg ISDN exhibited optimal mechanical stability, controlled drug release, and biocompatibility. These findings suggest that starch/ECM/ISDN scaffolds hold potential for wound healing applications. Further studies should focus on in vivo evaluation and cytotoxicity assessments to confirm their clinical applicability.

## 1. Introduction

Wound healing is a multifaceted biological process encompassing hemostasis, inflammation, proliferation, and tissue remodeling. The development of biomaterials that support and accelerate this process has garnered considerable interest in regenerative medicine [1]. Among these, biopolymeric scaffolds have emerged as viable alternatives for dermal wound care due to their inherent biocompatibility, degradability, and capacity to replicate the extracellular matrix (ECM) [2].

An ideal scaffold for wound healing should exhibit specific physicochemical and mechanical properties that foster cellular adhesion, proliferation, and differentiation. Structural characteristics such as high porosity, an interconnected network, and an adequate surface area-to-volume ratio enhance cellular migration and nutrient exchange [3]. Furthermore, flexibility and adaptability to the wound site are critical to ensuring proper integration and function. In addition to biocompatibility and controlled degradation aligned with the wound healing timeline, maintaining a moist microenvironment is essential for facilitating cellular attachment, enhancing angiogenesis, accelerating granulation tissue formation, and promoting re-epithelialization [4]. Functionalization with bioactive molecules further improves scaffold performance by stimulating cellular responses and expediting the healing process [5].

Among the various biopolymeric materials, starch-based scaffolds offer a promising alternative due to their abundance, biodegradability, and non-toxic nature. Their ability to form hydrogels allows for effective moisture retention, creating a suitable environment for cell adhesion and proliferation, making them attractive candidates for next-generation wound healing strategies [6].

Cassava starch (S) is widely recognized for its excellent film-forming and gelling properties, making it a suitable candidate for scaffold fabrication. However, its inherent brittleness and high hydrophilicity limit its mechanical performance and stability [5]. To overcome these challenges, the incorporation of decellularized ECM (dECM) can enhance the structural and bioactive properties of the scaffold, providing biochemical cues essential for cell migration, proliferation, and differentiation. dECM-derived scaffolds have been shown to promote angiogenesis and tissue regeneration due to the presence of growth factors and bioactive molecules that support the wound healing process [7].

Additionally, the incorporation of isosorbide dinitrate (ISDN), a nitric oxide donor with vasodilatory properties, can further enhance wound healing by promoting angiogenesis and improving blood flow to the injured tissue [8]. Nitric oxide plays a critical role in cellular signaling pathways involved in wound repair, modulating inflammatory responses, and accelerating tissue regeneration [9]. By integrating ISDN into a starch–dECM scaffold, a multifunctional biomaterial can be developed with enhanced mechanical stability, bioactivity, and controlled drug release capabilities.

This study aimed to synthesize and characterize a novel biopolymeric scaffold composed of cassava starch biofunctionalized with dECM and ISDN. Although similar starch-based systems have been synthesized [6,10,11], this combination has not been previously reported and represents a new composite system with enhanced bioactivity and therapeutic potential. We hypothesized that the combination of these components will provide the scaffold with optimal physicochemical properties, including suitable porosity, moisture retention, and mechanical stability, essential for supporting cellular adhesion, proliferation, and tissue regeneration.

## 2. Materials and Methods

### 2.1. Starch Extraction

A single batch of cassava starch was extracted following the methodology described by Linares-Bravo et al. [12]. Fresh cassava tubers were sourced from a local market and subjected to a thorough cleaning process using soap and water. The cleaned cassava was then weighed, peeled, and cut into uniform medium-sized pieces. These pieces were subsequently ground using a stainless-steel blade from a Sunbeam stick mixer (San Luis Potosí, SLP, México) to obtain a homogeneous pulp.

The resulting cassava pulp was initially filtered through a conventional strainer to remove coarse residues. To further refine the starch extraction, the filtrate was sequentially passed through a series of sieves with mesh sizes of 150, 75, and 38 µm, as outlined by Maniglia et al. [13]. The starch-containing suspension was then allowed to settle for 24 h to facilitate sedimentation. Following this, the precipitated starch was subjected to drying in a Quincy lab. Incubator (Chicago, IL, USA) model 12–140 at 45 °C for 8 h.

Once dried, the starch was finely ground using a mortar and pestle to achieve a uniform particle size and was subsequently stored in hermetically sealed containers at a controlled relative humidity of 35% until further use. Iodine colorimetry testing was performed to confirm starch presence and its amylose/amylopectin ratio. In addition, pH, moisture, and ash content analyses were performed to further characterize the cassava starch. The characterization results of the extracted cassava starch, including iodine colorimetry, moisture content, and ash content, are presented in Appendix A.

### 2.2. Decellularized ECM Obtention and ISDN Obtention

Decellularized ECM was obtained from porcine urinary bladders sourced from pigs at the San Luis Potosí Municipal Rastro, which is ISO 9001-2008-certified [14]. The bladders were rinsed in a 10% iodine solution for decontamination and transported in phosphate-buffered saline (PBS) with 100 IU/mL of penicillin and 100 mg/mL of streptomycin. They were mechanically delaminated to separate the inner layer of muscle, followed by decellularization. Once complete, the ECM was subjected to enzymatic digestion, frozen, and lyophilized for further use, as described by Kao et al. [15]. ISDN was obtained from Isorbid AP capsules from Armstrong Labs. De México (México City, México), which were opened and crushed in an agate mortar and then stored for later use.

### 2.3. Synthesis of Biopolymeric Systems

Four groups of biopolymer-forming solutions were prepared using the casting method, following a modified version of the methodology described by Cheng et al. [16]. Groups were established based on two variables: dECM content (7 and 12.5% *w*/*w*) and ISDN concentration (20 and 40 mg). The selection of dECM and ISDN concentrations in the biopolymeric systems was guided by evidence from previous studies. Specifically, the dECM content was chosen based on the work of Cuevas-Tapia et al. [17], who demonstrated the biocompatibility and bioactivity of porcine urinary bladder-derived ECM in tissue engineering applications. In our study, we tested formulations with lower ECM percentages to evaluate whether functionalization could still be achieved at reduced concentrations while maintaining similar efficacy. Regarding ISDN, the selected concentrations were informed by prior studies [18,19], where lower doses of this compound were effectively employed for localized biomedical applications. Considering that the biopolymeric system is expected to remain in contact with the wound for several days and provide sustained release, the ISDN amount was adjusted accordingly to ensure therapeutic relevance over the intended application period. The final compositions are detailed in Table 1.

**Figure 1:**
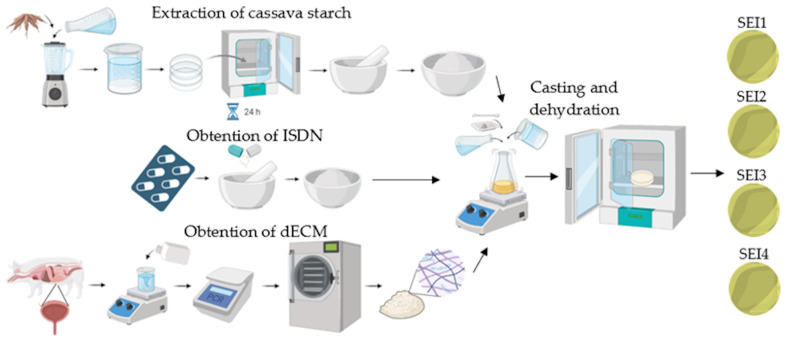
Schematic illustration of the obtention process of the biopolymeric systems’ components and subsequent synthesis.

For the preparation of the scaffolds, the casting method was used in order to obtain thin films. Starch (5% *w*/*v*) was poured into an aqueous suspension of distilled water, with Sigma-Aldrich glycerol (St. Louis, MO, USA) (30% *w*/*w*) added as a plasticizer and citric acid (1% *w*/*w*) from Sigma-Aldrich (St. Louis, MO, USA) as a crosslinking agent. The mixture was stirred in a Lab companion (Daejeon, Republic of Korea) HP-3100 stirrer at 250 rpm for 15 min at 90 °C. Subsequently, dECM was added and was stirred for 15 min; then, the temperature was decreased to 70 °C and ISDN was added to the mixture, and it was stirred for 10 min until gelation. The molding method was used for the formation of biopolymeric systems; 48 mL of biopolymeric forming suspension was poured in a circular mold 8.42 cm in diameter and with a 55.65 cm^2^ capacity, and the systems were dehydrated in an Arsa AR-130 incubator (Zapopan, JAL, México) at 35 °C for 48 h before demolding and storage. The synthesis process of the biopolymeric systems is schematically shown in Figure 1.

### 2.4. Physicomechanical Characterization

#### 2.4.1. Swelling and Degradation Test

Swelling and degradation tests were evaluated in quadruplicate. Swelling measurements were taken at intervals of up to 15 days, while degradation was assessed for 30 days. To evaluate the swelling and degradation of the biopolymeric systems, static tests were performed. Samples of the different biopolymeric systems were weighed ($W_{s}$) and immersed in simulated body fluid (SBF) with a quantity of 2.5 mg/mL of SBF. Samples were placed in a 24-well box and incubated statically at 37 °C in an Arsa AR-130 incubator, Feligneo (Zapopan, JAL, México) for each measurement time. Once the time for each interval had elapsed, the samples were removed from the incubation with the help of a spatula and the excess humidity was removed with a Whatman filter paper, Whatman Plc (Maidstond, Kent, United Kingdom). Samples were weighed in an Ohaus analytical balance (México City, México), and their mass was recorded ($W_{w}$) [20]. Swelling and degradation were calculated according to the following equation:

(1) $$Swelling\;degree\;=\frac{W_{w}-W_{s}}{W_{w}}\times 100$$

#### 2.4.2. Porosity

Porosity was evaluated using the ethanol saturation method under vacuum conditions, allowing for the quantification of interconnected voids within the material. The analysis was performed in quadruplicate to ensure reproducibility. Samples were cut into 1 cm^2^ squares and placed in a desiccator under vacuum to remove any residual air within the porous structure. Subsequently, the samples were immersed in absolute ethanol and maintained under vacuum conditions for a 24 h period to ensure complete infiltration of the solvent into the material’s internal voids, as described by Adeli et al. [21]. After saturation, the excess ethanol was carefully removed, and the samples were weighed to determine the absorbed solvent content. Porosity was calculated using the following equation:

(2) $$Porosity\;(\%)=\frac{W_{1}-W_{0}}{\rho_{ethanol\;}\times V}\times 100$$

where $W_{1}$ is the final weight of the biopolymeric system after being immersed in ethanol, $W_{0}$ is the initial weight of the dry biopolymeric system, $\rho_{ethanol\;}$ is the ethanol density, and V is the volume of the dry system.

#### 2.4.3. Moisture Permeability and Porosity

Water vapor transmission rate (WVTR) tests were conducted in quadruplicate by following a modified ASTM E96 standard [22]. Samples were cut into circular specimens with a 1.5 cm diameter matching the opening of the test tube and were conditioned at room temperature (25 ± 2 °C) and 50% relative humidity before testing. Each sample was placed at the mouth of a test tube containing a pre-measured amount of distilled water. The edges of the tube were sealed with micropore tape to prevent lateral water loss and ensure that water vapor diffusion occurred only through the material. The assembled system was incubated in an Arsa AR-130 incubator (Zapopan, JAL, México) containing a controlled humidity environment. The initial weight of each tube was recorded, and weight measurements were taken at regular intervals over a 72 h period using an analytical balance with a precision of 0.1 mg. The WVTR was calculated using the following equation:

(3) $$Water\;vapor\;transmission\;rate\;(\frac{\frac{g}{m^{2}}}{day})=\frac{W_{i}-W_{f}}{A}\times 100$$

where $W_{f}$ is the final weight of the biopolymeric system after being measurement times, $W_{i}$ is the initial weight of the biopolymeric system, and A is the area of the mouth of the tube.

#### 2.4.4. Mechanical Characterization

The tensile testing method was carried out in quadruplicate to evaluate the mechanical properties of the scaffolds. The samples were cut into 20 × 10 mm rectangles and subjected to a tensile test, as recommended by the ASTM D638-14 Standard Test Method [23]. The test was performed on a Shimadzu universal testing machine (UTM) model AGS-X (Montevideo, Uruguay) with a 500 N load cell and a speed of 10 mm/min [24].

### 2.5. Physicochemical Characterization

#### 2.5.1. Fourier Transform Infrared (FTIR) Spectroscopy

FTIR was performed to analyze the chemical composition and possible interactions between starch, dECM, and ISDN. Samples were sterilized with UV light and analyzed on an Agilent Technologies Cary 600 spectrometer (Santa Clara, CA, USA), using the attenuated reflection technique (ATR) with 32 scans at a 4 cm^−1^ resolution in the range of 525 to 4000 cm^−1^. The data obtained were analyzed with the OMNIC Paradigm software version 2.7 from Thermo Scientific and OriginPro software version 8.5 [25].

#### 2.5.2. Thermogravimetric Analysis

Thermogravimetric analysis (TGA) was performed to evaluate the thermal stability and degradation profile of the scaffolds. Approximately 10 mg of each sample was weighed and introduced into a TA Instruments TGA Q500 (New Castle, DE, USA). The analysis was performed under an inert nitrogen atmosphere with a flow rate of 10 mL/min, in a temperature range from 50 to 600 °C at a rate of 10 °C/min [26].

### 2.6. Morphological Characterization

Morphological characterization was carried out using scanning electron microscopy (SEM) (JEOL model JSM6510V; Akishima, Tokyo, Japan) at an accelerated voltage of 5 kV to examine the surface topography and internal structure of the material. Sample preparation methods included sputter-coating with gold for 25 s at 4 mA under vacuum conditions.

### 2.7. ISDN Release

A calibration curve for ISDN quantification was prepared by measuring the absorbance of standard ISDN solutions at 220 nm. Standards were prepared at various concentrations to establish a linear relationship between concentration and absorbance. The release test was carried out in quadruplicate. Samples were prepared by taking known quantities of the biopolymeric systems and then immersing them in SBF at room temperature to simulate physiological conditions. The samples were maintained statically in a 24-well plate. A starch film without ISDN was used as a control. At predetermined time intervals, 1000 µL aliquots of the release medium were withdrawn using a micropipette. The absorbance of each aliquot was measured using a Varian Cary 50 Bio UV-Vis spectrophotometer, Agilent Technologies Inc. (Palo Alto, CA, USA) set to 220 nm [27]. The concentration of ISDN in each aliquot was determined using the calibration curve. The concentration values were used to calculate the cumulative amount of ISDN released over time. The cumulative release data were plotted against time to create a release profile.

### 2.8. Hemocompatibility

The hemolysis assay was performed following the guidelines outlined in ISO 10993-4:2017 [28], which specifies methods for evaluating the interactions of medical devices with blood. This assay was used to assess the potential erythrocyte damage induced by the biopolymeric systems based on hemoglobin release quantification. Fresh human blood was obtained from a healthy donor and collected in EDTA-treated anticoagulant tubes. Positive and negative control solutions were prepared to validate the assay’s accuracy. The red blood cells (RBCs) were then exposed to the biopolymeric systems in test tubes, prepared in quadruplicate, and incubated at room temperature at various time intervals from 15 min to 24 h, following the methodology described by Weber et al. [29].

Following incubation, the samples were centrifuged at 5000 rpm for 10 min in an Eppendorf 5804 R centrifuge (Hamburg, Germany) to separate the plasma containing the lysed hemoglobin from intact RBCs. The hemoglobin concentration in the supernatant was quantified by measuring the absorbance at 540 nm using a Thermo Scientific Multiskan FC plate reader (Waltham, MA, USA). The amount of hemoglobin released was directly correlated with the degree of hemolysis, allowing for the assessment of the hemocompatibility of the biopolymeric systems.

### 2.9. Statistical Analysis

The Shapiro–Wilk test was used to assess the normality of the data. For datasets that met the normality assumption, a one-way analysis of variance (ANOVA) was performed to determine significant differences among groups. In contrast, for datasets that did not pass the normality test, Kruskal–Wallis one-way analysis of variance was performed. All statistical analyses were conducted using the SigmaPlot program version 11.0, with a significance level set at *p* < 0.05.

## 3. Results

Four different biopolymeric systems and a starch-only control membrane were obtained in circular films with an approximate thickness of 0.5 ± 0.04 cm and an 8 ± 0.05 cm diameter and a sepia-like color. These systems were evaluated to determine their properties.

### 3.1. Physicomechanical Characterization

#### 3.1.1. Swelling and Degradation Test

**Figure 2:**
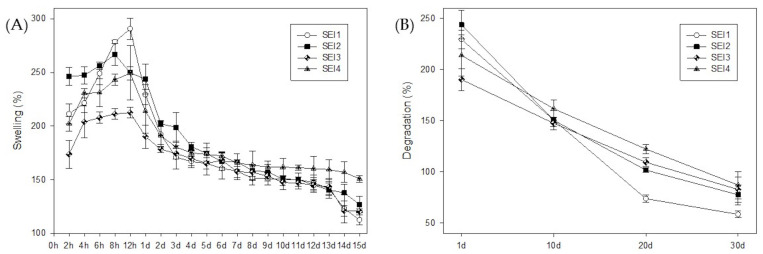
Swelling and degradation behavior of the biopolymeric systems. (**A**) Swelling profile from 2 h to 15 days. The initial section (first quadrant) shows data in hourly increments to highlight the maximum swelling observed between 8 and 12 h, followed by daily measurements from day 1 to day 15. (**B**) Degradation profile presented separately, with data recorded at 10-day intervals to provide a clearer view of the long-term degradation behavior.

All biopolymeric systems exhibited swelling percentages greater than 100% during the first 15 days, demonstrating their high water absorption capacity. The swelling behavior followed a characteristic trend over time, as shown in Figure 2. Initially, all formulations underwent a rapid increase in swelling within the first 8–12 h, reaching their maximum values within this period. Biopolymeric system SEI1 showed the greater swelling rate at 12 h, but it also showed the greater weight loss at day 30. In contrast, the control membrane (S) exhibited its highest swelling at approximately 167% on day 2, and by day 7, its swelling percentage had already decreased to levels comparable to those observed in the biopolymeric systems at day 30. The swelling profile of the control (S) is presented in Appendix B.

Among the evaluated systems, differences in degradation rates were observed. SEI2 and SEI3 showed a more rapid reduction in swelling after day 7, indicating accelerated degradation, possibly due to higher porosity or a lower degree of crosslinking occasioned by differences in formulation concentrations. Others maintained a stable structure for a more extended period, until day 13 approximately, degrading more gradually until day 30, which suggests greater resistance to dissolution. This degradation behavior is crucial for wound healing applications, as it enables controlled bioactive release and the gradual disappearance of the biomaterial in sync with tissue regeneration.

#### 3.1.2. Porosity

**Figure 3:**
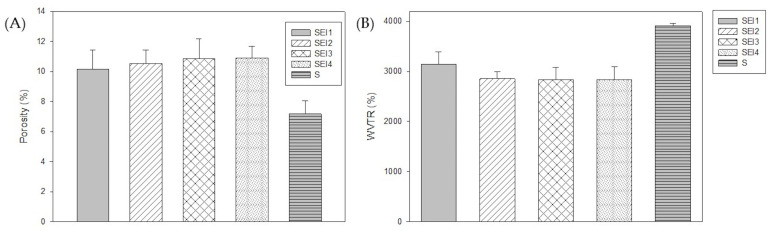
(**A**) Porosity percentage of biopolymeric systems at 24 h and (**B**) WVTR of biopolymeric systems at 72 h.

The porosity results, shown in Figure 3, indicate that SEI1, SEI2, SEI3, and SEI4 exhibited similar porosity values ranging from approximately 10 to 12%. In contrast, the starch (S) membrane used as a control demonstrated a significantly lower porosity value (~7.2%), suggesting a denser structure. The ANOVA results indicated a statistically significant difference among the groups (*p* = 0.007). To further analyze these differences, a Tukey multiple-comparison test was performed. The results revealed significant differences between the control group (S) and SEI formulations.

#### 3.1.3. Moisture Permeability

The WVTR analysis revealed that SEI1, SEI2, SEI3, and SEI4 exhibited similar permeability levels, averaging around 3000 g/m^2^/day, which is within the desirable range for wound healing materials [4]. Notably, SEI1 showed the highest WVTR, as shown in Figure 3, exceeding 3000 g/m^2^/day, despite having the lowest porosity, suggesting that other structural factors may contribute to its permeability behavior. A starch membrane with micropore tape (S) was evaluated as a control. It displayed significantly higher WVTR values, exceeding 3500 g/m^2^/day, indicating an enhanced capacity for moisture exchange in comparison with the biopolymeric systems. The ANOVA revealed no statistically significant differences among the treatment groups (*p* = 0.266), indicating that variations in WVTR values across the biopolymeric systems were likely due to random sampling variability rather than inherent differences in material composition.

#### 3.1.4. Mechanical Characterization

The mechanical characterization of the biopolymeric systems revealed variations in stress, strength, strain, and Young’s modulus, as shown in Table 2. Among the tested formulations, SEI3 exhibited the highest strength (4.16 ± 0.29 N) and a high stress value (0.41 ± 0.02 N/mm^2^), indicating improved load-bearing capacity. SEI1 also demonstrated good mechanical performance (3.63 ± 0.10 N, 0.36 ± 0.01 N/mm^2^), whereas SEI2 and SEI4 showed lower resistance under tensile force, with strength values of 2.50 ± 0.19 N and 2.30 ± 0.16 N, respectively. In terms of strain, SEI2 displayed the highest elongation (19.64 ± 0.89%), suggesting it is the most flexible formulation. SEI1, SEI3, and SEI4 showed intermediate strain values (13.59% to 15.52%), while the control system (S) had the lowest deformation (12.45 ± 0.44%). This indicates that although none of the systems are particularly rigid, the control is slightly less flexible compared to the biofunctionalized systems. When evaluating stiffness through Young’s modulus, the control system (S) exhibited the highest value (3.62 ± 0.11 MPa), followed by SEI1 and SEI3 (both at 2.68 MPa). In contrast, SEI2 and SEI4 had the lowest modulus values (1.27 ± 0.05 MPa and 1.55 ± 0.10 MPa), reflecting their increased elasticity. Overall, these results suggest that SEI3 offers the most favorable balance between mechanical strength and moderate flexibility among the biopolymeric systems. A Kruskal–Wallis one-way ANOVA on ranks revealed no statistically significant differences among the formulations (*p* = 0.722), suggesting that variations in mechanical properties were likely due to random sampling variability rather than inherent differences in material composition.

### 3.2. Physicochemical Characterization

#### 3.2.1. Fourier Transform Infrared (FTIR) Spectroscopy

**Figure 4:**
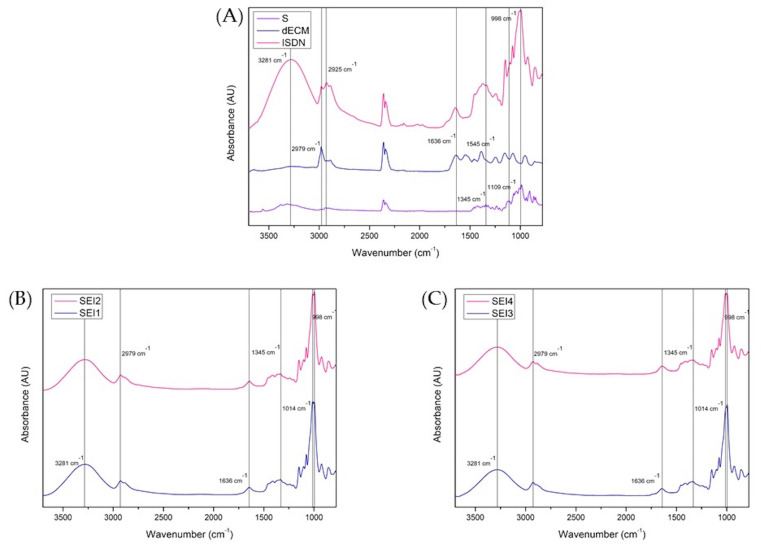
FTIR spectra recorded in the range of 3750 to 750 cm^−^^1^ for (**A**) S, dECM, and ISDN; (**B**) SEI1 and SEI2; and (**C**) SEI3 and SEI4. A broad band at 3281 cm^−^^1^ corresponding to O–H stretching can be observed in all samples, indicating the presence of starch. A band at approximately 1636 cm^−^^1^ can be observed in all formulations. This band is typically associated with the Amide I vibration of proteins, suggesting the presence of dECM. However, it is important to note that this region may also be influenced by contributions from starch, so the assignment of this band should be interpreted with caution, as it may reflect overlapping signals from both starch and dECM. The peaks observed at approximately 1345, 1420, and 1463 cm^−^^1^ may arise from both ISDN and starch-related functional groups, as there is considerable overlap between the signals from starch and ISDN in this region. Therefore, the assignment of these peaks is uncertain and should be interpreted with caution, as they likely reflect a combination of contributions from both starch and ISDN.

The Fourier transform infrared (FTIR) spectra of the individual components and the biopolymeric systems SEI1, SEI2, SEI3, and SEI4 are presented in Figure 4. The analysis confirmed the presence of key functional groups and molecular interactions within the biopolymeric matrices. The spectra of the pure components exhibited characteristic absorption bands, with starch displaying a strong O-H stretching vibration at 3281 cm^−^^1^ and C-O-C stretching at 998 cm^−^^1^. The dECM displayed a distinctive Amide I band at 1636 cm^−^^1^, as well as a band at 2979 cm^−^^1^ associated with C-H stretching present in both starch and dECM. Peaks observed at 1345, 1420, and 1463 cm^−^^1^ may be associated with the NO_2_ functional groups of ISDN, suggesting its potential presence within the matrix [30]. However, due to the structural similarities between starch and ISDN and the predominance of starch in the system, it is likely that these signals overlap, partially or fully masking ISDN-specific peaks.

In the biopolymeric systems, these functional groups were detected with slight shifts and variations in intensity, indicating molecular interactions between the components. The comparative analysis of SEI1-SEI2 and SEI3-SEI4 revealed compositional similarities, while shifts in the hydroxyl and amide regions suggested potential hydrogen bonding and structural rearrangements. These findings underscore the influence of formulation composition on the microstructural characteristics of the biopolymeric matrices, which may subsequently impact their functional properties in biomedical applications.

#### 3.2.2. Thermogravimetric Analysis

**Figure 5:**
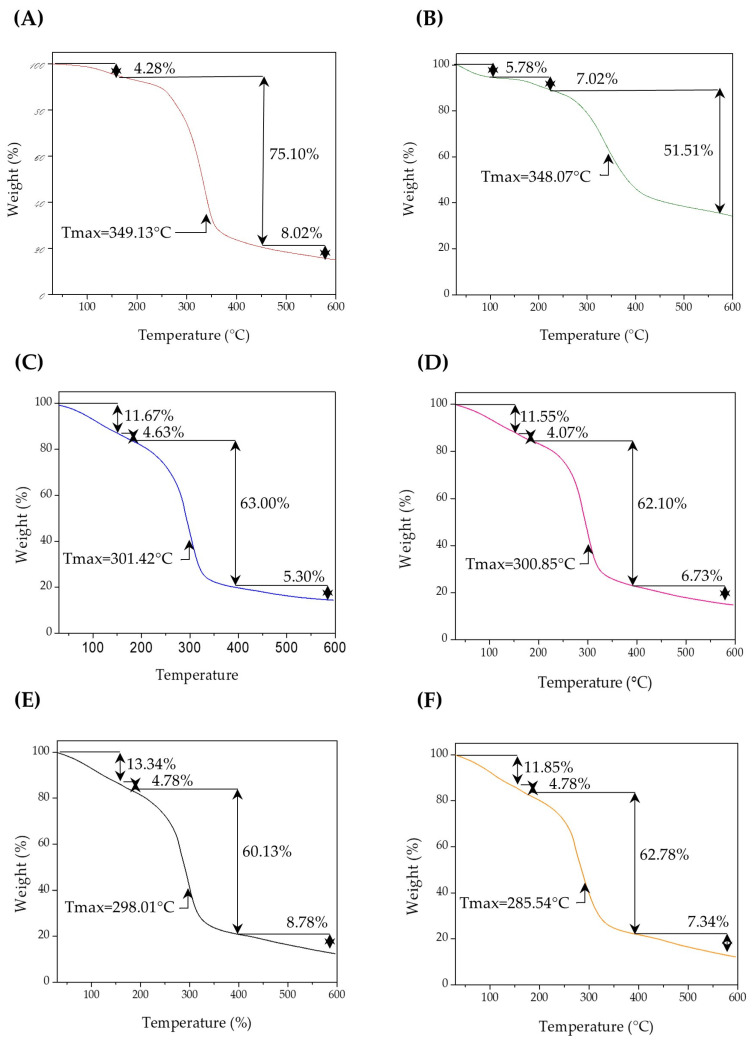
Thermograms for (**A**) S, (**B**) dECM and biopolymeric systems, (**C**) SEI1, (**D**) SEI2, (**E**) SEI3, and (**F**) SEI4, where an initial constant degradation can be observed in all biopolymeric systems between 50 and 150 °C, followed by a simple degradation step between 180 and 400 °C, with a weight loss of approximately 52–63%.

The thermogravimetric analysis (TGA) of the biopolymeric systems SEI1, SEI2, SEI3, and SEI4 revealed similar thermal degradation profiles, as shown in Figure 5, characterized by three main stages of weight loss. The initial mass loss, occurring between 50 and 150 °C, corresponds to the evaporation of residual moisture and volatile compounds (~11–13 wt%), with SEI4 showing the highest water content. The primary decomposition stage was observed between 180 and 400 °C, with a weight loss of approximately 52–63%, attributed to the pyrolysis of amylose and amylopectin in starch, generating water, carbon dioxide, carbon monoxide, acetaldehyde, furan, and 2-methylfuran [31]. This degradation is likely due to the breakdown of starch, extracellular matrix, and isosorbide dinitrate, given their organic nature. It is also related to the interaction between plasticizers and starch [32]. Two peaks observed in the derivative during the main decomposition step may be associated with interactions involving dECM and ISDN.

When compared to the thermal profiles of the individual components—S and dECM—notable differences were observed. The control membrane (S) exhibited a significant degradation beginning around 270 °C and a major weight loss peak around 350 °C, corresponding to the thermal degradation of saccharides. In contrast, dECM presented a broader degradation pattern, with significant weight loss beginning around 300 °C and a peak near 400 °C. This behavior reflects the complex composition of the extracellular matrix, which includes collagen and other proteinaceous components. In contrast, the SEI systems exhibited intermediate thermal behaviors, with degradation events spanning both ranges, indicating the integration of both components. Among the materials, SEI1 and SEI2 exhibited slightly higher thermal stability than SEI3 and SEI4. This could be attributed to the lower concentration of ISDN and dECM in SEI1 and SEI2, which may enhance the bonding between the plasticizers and starch. Consequently, the interaction between the citric acid–glycerol system and starch appears to be stronger in the presence of dECM than ISDN. The final degradation stage, occurring beyond 400 °C, involved the breakdown of carbonaceous residues, leaving approximately 12.71 to 14.87 wt% of the initial mass as residue [33].

Notably, SEI1 and SEI2 showed higher residual mass percentages than SEI3 and SEI4, particularly when the ISDN content was 40 mg. The minor differences in thermal stability among the samples suggest that formulation composition affects the degradation kinetics, although these changes do not appear to significantly impact the thermal performance of the biopolymeric systems for biomedical applications.

### 3.3. Morphological Characterization

**Figure 6:**
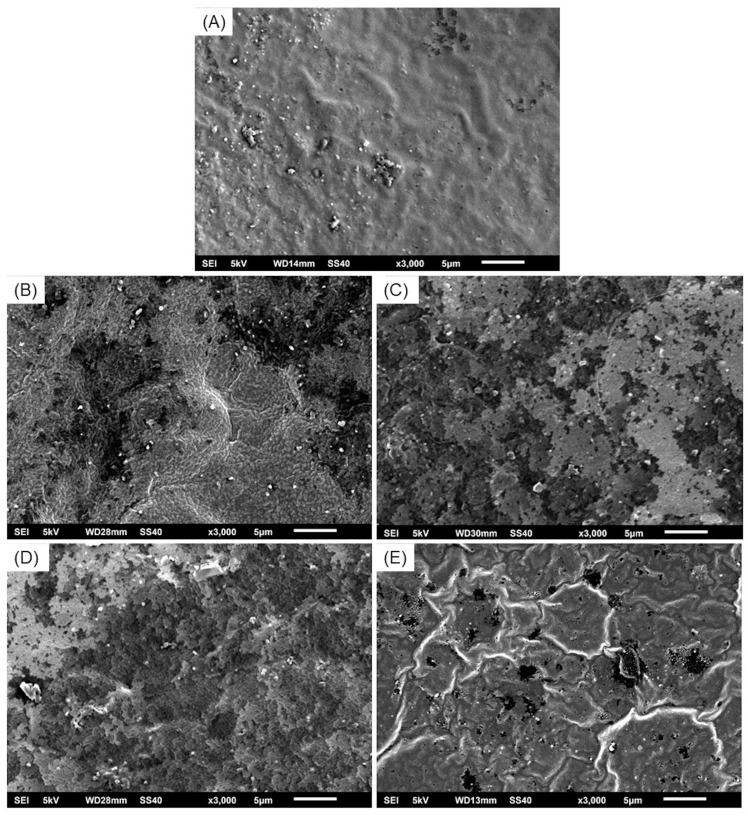
Micrographs at ×3000 magnification of (**A**) S, where a continuous and homogeneous surface can be observed; (**B**) SEI1, (**C**) SEI2, and (**D**) SEI3, where layered structures can be observed; and (**E**) SEI4, where a porous structure can be observed with less visible layers.

The scanning electron microscopy (SEM) analysis of the biopolymeric systems revealed a generally rough topography across all formulations, though no significantly large pores were observed. As shown in Figure 6, SEI1 and SEI2 exhibited relatively smooth surfaces with minor irregularities, likely associated with starch crystallization. These two systems also displayed more compact and homogeneous morphologies, suggesting improved structural integrity and polymeric cohesion, but reduced porosity. In contrast, SEI3 and SEI4 showed more heterogeneous and fractured textures, with visible layered structures and evidence of phase separation. The control membrane (S) presented a distinct surface morphology compared to the biofunctionalized systems. It showed a more homogeneous and continuous surface without any visible porosity or layered structure. However, some undissolved starch granules were visible, indicating incomplete gelatinization or dispersion during processing. Despite these granules, the surface appeared cohesive and uninterrupted, which contrasts with the stratified appearance of the modified formulations. The increased porosity observed in SEI3 and SEI4 may influence their mechanical and degradation properties, potentially impacting their performance in controlled-release applications [34]. These findings indicate that formulation composition significantly affects the microstructural characteristics of the biopolymeric systems.

### 3.4. ISDN Release

**Figure 7:**
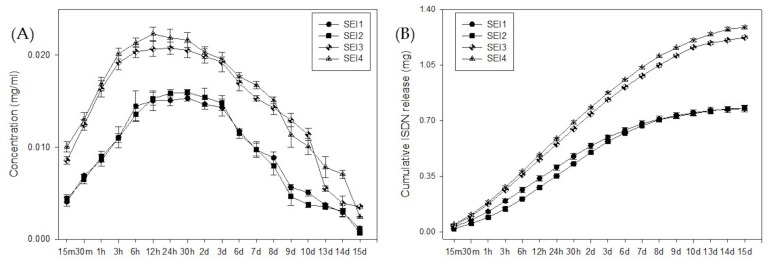
ISDN (**A**) concentration release and (**B**) cumulative release from biopolymeric systems over 15 days.

The release kinetics of ISDN from the biopolymeric systems were evaluated over a 15-day period. In Figure 7, the concentration–time profile is shown (left) presenting a biphasic release behavior, with an initial burst phase during the first 24 h, followed by a sustained release phase. SEI1 and SEI4 exhibited higher initial release rates compared to SEI2 and SEI3, suggesting differences in matrix composition and porosity. The cumulative release profile (right) indicated that SEI1 and SEI4 reached a higher total ISDN release (~1.3 mg) than SEI2 and SEI3 (~0.9 mg), confirming that the formulation influences drug diffusion. To determine the release mechanism, the data were fitted to common drug release models, including zero-order, first-order, Higuchi, and Korsmeyer–Peppas models [35,36]. The best fit was obtained with the Korsmeyer–Peppas model (R^2^ > 0.98), indicating a non-Fickian or anomalous transport mechanism, where both diffusion and polymer relaxation contribute to drug release.

The Korsmeyer–Peppas model describes drug release from a polymeric system.

(4) $$Qt=K_{KPt}^{n}$$

where $K_{KP}$ is the Korsmeyer–Peppas constant and *n* is the release exponent describing the drug release mechanism. These results suggest that the structural characteristics of the biopolymeric matrices significantly modulate ISDN release, making them potential candidates for controlled drug delivery applications.

### 3.5. Hemocompatibility

**Figure 8:**
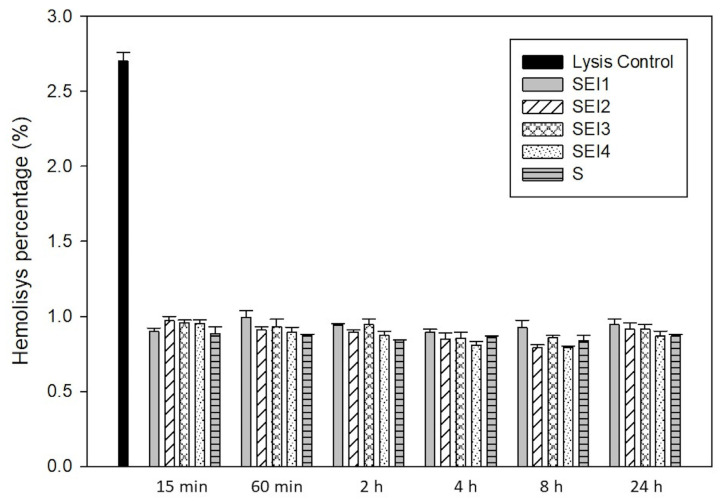
Hemolysis percentages of RBCs in contact with biopolymeric systems SEI1–4 and control membrane (S) after 15 and 60 min and 2, 4, 8, and 24 h.

The hemocompatibility of the biopolymeric systems was evaluated through a hemolysis assay over a 24 h period. As shown in Figure 8, all tested formulations—SEI1, SEI2, SEI3, SEI4, and the control (S)—exhibited hemolysis percentages consistently below 1.0% at all measured time points. These values fall well within the internationally accepted threshold (<2%) for materials intended for biomedical applications, indicating that the systems do not cause significant red blood cell membrane disruption. Notably, the control system (S) also demonstrated low hemolytic activity comparable to the biofunctionalized systems, supporting its biocompatibility. In contrast, the positive lysis control showed a significantly elevated hemolysis percentage (~2.7%) at the 15 min mark, confirming the validity and sensitivity of the assay. The ANOVA results indicated a statistically significant difference among groups (F = 137.198, *p* < 0.001), suggesting that variations in hemolysis levels were not due to random sampling variability. These findings indicate that the SEI biopolymeric systems demonstrate excellent hemocompatibility, making them suitable for biomedical applications, including controlled drug release and tissue engineering [37].

## 4. Discussion

The present study highlights the potential of starch/dECM/ISDN biopolymeric systems for biomedical applications, particularly in drug delivery and tissue engineering. The observed swelling and degradation behaviors provide critical insights into the suitability of these formulations for maintaining an optimal wound environment and ensuring sustained therapeutic efficacy. All formulations demonstrated a high swelling capacity, exceeding 100% within the first 15 days, a characteristic that is essential for maintaining a moist environment conducive to tissue regeneration [4,20]. The swelling trend followed a biphasic pattern, with a rapid hydration phase within the first 8–12 h, followed by a gradual decline over time (Figure 1). The initial rapid fluid uptake can be attributed to the hydrophilic nature and porous structure of the biopolymeric matrices, which facilitate efficient moisture retention and diffusion. This finding aligns with previous research highlighting the importance of moisture in promoting effective wound healing [38].

SEI1 exhibited the highest swelling rate at 12 h, suggesting an enhanced ability to absorb and retain moisture. These results coincide with the WVTR results, with SEI1 being the biopolymeric system with the highest WVTR. However, SEI1 also showed the greatest weight loss by day 30, suggesting that its structural integrity is more susceptible to aqueous degradation. This accelerated breakdown is possibly attributed to a lower crosslinking degree or increased solubility of its components, factors that have been previously linked to polymer stability and degradation kinetics [39]. In contrast, the control membrane (S) exhibited a consistently lower swelling percentage compared to the biopolymeric systems at all evaluated time points. Notably, by day 7, the swelling of membrane S reached levels comparable to those observed in the biopolymeric membranes at day 30. This behavior may be associated with its morphology, as evidenced by the SEM images in Figure 6, which reveal a smooth and homogeneous surface lacking visible layers or pores. This contrasts with the biopolymeric systems, where stratified structures were evident in SEI1, SEI2, and SEI3 and porous features were observed in SEI4. Such structural characteristics likely enhance water absorption capacity, contributing to the higher swelling performance of the biopolymeric membranes. These morphological observations are further supported by the porosity test results, in which the control membrane (S) showed significantly lower porosity percentages than those recorded for the SEI systems, reinforcing the correlation between porous architecture and swelling behavior. The differential degradation rates among formulations further support the role of structural and compositional variations in influencing material longevity. SEI2 and SEI3 experienced a more rapid decline in swelling after day 7, indicating an accelerated degradation process likely due to higher porosity or reduced crosslinking density, which facilitates water infiltration and polymer breakdown [40]. Nevertheless, further studies incorporating structural analysis—such as FTIR for ester bond identification, swelling behavior quantification, or solid-state NMR—would be essential to confirm the exact nature and extent of the crosslinking in these systems. In contrast, SEI4 maintained structural integrity longer, remaining stable until approximately day 13 and degrading more gradually up to day 30. This suggests that SEI4 possesses greater resistance to dissolution, making it a promising candidate for controlled drug release and durable wound dressings [38]. The gradual decline in swelling over time across all formulations indicates a controlled degradation process. Such behavior is essential for biomedical applications, particularly in drug delivery and tissue engineering, where a predictable degradation profile ensures sustained therapeutic effects and scaffold support during tissue regeneration [41]. Materials that maintain stability for at least 10 days before initiating degradation are ideal for wound healing applications, as they provide sufficient time for bioactive release and tissue repair without premature scaffold breakdown [39].

SEI1, SEI2, SEI3, and SEI4 exhibited water vapor transmission rates (WVTRs) averaging approximately 3000 g/m^2^/day at day 3. This value fits the optimal range suggested by some studies for wound dressings, which typically falls between 800 and 1000 g/m^2^/day. However, other researchers have reported that a WVTR of approximately 2028 g/m^2^/day is beneficial for wound healing [42]. Thus, SEI formulations are suitable for maintaining an optimal moist environment for wound healing. It is particularly interesting that SEI1 exhibited the highest WVTR, exceeding 3000 g/m^2^/day, despite having the lowest porosity among the SEI formulations. This observation is consistent with the results obtained for the control membrane (S), which exhibited the highest WVTR value. Considering that SEI1 is the formulation with the lowest contents of ECM and ISDN, its behavior aligns with that of the membrane composed solely of S, suggesting that the presence of S plays a predominant role in facilitating water vapor transmission regardless of overall porosity. This finding suggests that additional structural factors, such as polymer crosslinking density, pore interconnectivity, and hydrophilicity, may influence vapor permeability. Previous studies have indicated that crosslinked biopolymeric networks can enhance moisture transport efficiency by promoting hydrophilic interactions, even when porosity is relatively low [43]. A more porous structure in the SEI biopolymeric materials may enhance moisture retention, facilitate cellular infiltration, and improve gas exchange, all of which are desirable characteristics for wound dressings [39]. Maintaining an optimal balance between moisture retention and breathability is essential for wound healing. Excessive porosity may lead to rapid fluid loss, while low porosity may result in exudate accumulation and an increased risk of infection [44].

Micrographs from SEI show the morphology and size of the pores in the systems, and these coincide with the ones taken by Linares-Bravo et al. [12] for cassava starch films. Formulations with smoother and less porous surfaces tend to exhibit greater polymeric cohesion and a more uniform distribution of biopolymeric components. This dense and homogeneous structure may be associated with stronger intermolecular interactions, potentially enhancing mechanical properties and reducing degradation rates [25]. SEI3 and SEI4 present a more fractured and porous structure, with evident phase separation, suggesting a less cohesive matrix. These morphological characteristics may result from variations in the distribution of components such as starch, extracellular matrix, and isosorbide dinitrate, which could negatively influence the material’s structural integrity. These results support the findings of the swelling and degradation tests. Despite SEI3 having a fractured structure, it exhibited the highest stress (~0.45 N/mm^2^) and strength (~4.5 N), suggesting superior load-bearing capacity compared to the other formulations. This finding indicates that SEI3 could be an optimal choice for applications requiring higher mechanical resilience, such as biodegradable structural components [25]. SEI1 also demonstrated notable mechanical properties, making it a suitable alternative for applications demanding moderate strength and stiffness. In terms of mechanical performance, the results did not show significant variation compared to the control membrane (S). Although the elasticity of membrane S was slightly lower, it remained within the same range as the SEI systems, indicating comparable flexibility and structural integrity across all formulations.

Although a fully defined chemical structure of the biopolymeric systems SEI1, SEI2, SEI3, and SEI4 is not yet available, the formulation and component interactions can be discussed based on their known molecular features and the preliminary spectroscopic data obtained. The starch provides a polysaccharide backbone capable of forming hydrogen bonds and physical entanglements. The dECM contributes proteinaceous components such as collagen, which may interact with starch primarily through hydrogen bonding. ISDN, a small-molecule nitrate donor, may become physically entrapped within the matrix or interact weakly via van der Waals forces and possible hydrogen bonding with hydroxyl groups of starch [30,45]. Spectroscopic analysis via FTIR confirmed the successful integration of starch, ECM, and ISDN within the polymeric matrices. The observed shifts in FTIR spectra and the respective intensity variations in specific absorption bands suggest molecular interactions, possibly due to hydrogen bonding and structural modifications [46]. The strong O-H stretching observed at 3281 cm^−^^1^ in starch, as well as the C-O-C stretching at 998 cm^−^^1^, were present in the biopolymeric systems, albeit with slight shifts. These spectral changes may indicate interactions between the hydroxyl groups of starch and other components, contributing to the overall stability of the polymeric matrix [33]. Further studies using complementary techniques such as differential scanning calorimetry (DSC) and X-ray diffraction (XRD) and molecular modeling could provide additional confirmation of chemical structure, molecular interactions, and crystallinity changes in these systems.

Thermal stability analysis via TGA indicated a three-stage degradation pattern across all formulations, consistent with prior findings on starch-based polymeric systems. The initial weight loss between 50 and 150 °C was attributed to the evaporation of residual moisture and volatile compounds, a phenomenon commonly observed in biopolymeric materials and reported to occur in extracellular matrix scaffolds [33]. The primary degradation phase (250–350 °C) corresponded to significant mass loss due to the breakdown of starch, dECM, and ISDN components. This observation aligns with previous studies reporting similar thermal behavior in starch-based polymeric systems, where degradation in this temperature range is primarily associated with the breakdown of glycosidic linkages and protein denaturation [47]. SEI1 exhibited slightly higher thermal stability, potentially due to its lower ISDN and dECM contents, supporting the notion that these organic additives influence thermal degradation kinetics. The presence of these organic components has been reported to influence the thermal degradation kinetics of polymeric matrices, with higher dECM and ISDN contents potentially leading to earlier decomposition due to their lower thermal resistance [33]. The thermogram of the control (S) membrane shows a main degradation step around 270 °C, which coincides with the primary thermal degradation observed in the biopolymeric systems. However, the initial gradual degradation is less pronounced compared to the SEI systems and the dECM thermograms. This suggests that the initial weight loss between 50 and 150 °C may be primarily attributed to moisture loss from the dECM, as previously reported by Liu et al. [45]. The main degradation peak of dECM appears around 400 °C, whereas in the S thermogram it occurs at approximately 350 °C, and in the SEI systems it can be observed around 300 °C. This shift could be attributed to alterations in polymer chain organization due to the incorporation of dECM. Similar findings were reported by Valencia-Llano et al. [11], who studied a system composed of cassava starch and chicken gelatin. Both dECM and chicken gelatin have a proteinaceous nature, and in both cases the main degradation peak occurred near 300 °C.

The residual mass variations suggest that formulation differences marginally impact ultimate thermal stability, confirming that these biopolymeric systems are thermally robust for biomedical applications [2]. Studies of biopolymeric systems composed of starches and proteinaceous components have shown similar results, with residual masses between 15% and 20%. This behavior has been attributed to the addition of gelatin, which affects the polymer chain arrangement and promotes the formation of more crosslinked structures, resulting in greater thermal stability as the temperature increases [11,48]. These findings are comparable to our observations of dECM-containing systems, where the presence of proteinaceous material may similarly influence the structural organization and enhance thermal stability through increased crosslinking. In particular, SEI1 and SEI2, which contain lower concentrations of dECM and ISDN, respectively, exhibited higher residual masses and greater thermal stability compared to SEI3 and SEI4. This suggests that moderate incorporation of dECM and ISDN may favor the formation of more thermally stable networks, consistent with the stabilizing effect observed in other starch–protein systems.

The incorporation of ISDN within the polymeric systems holds significant therapeutic potential, particularly in wound management. ISDN’s vasodilatory effects enhance angiogenesis by promoting capillary formation, a crucial factor in tissue repair [49]. Clinical studies have demonstrated a significant reduction in ulcerated areas upon ISDN application, reinforcing its relevance in advanced wound care [8]. Additionally, its anti-inflammatory properties contribute to a favorable wound healing environment by modulating immune responses and reducing oxidative stress [50]. The biphasic release pattern observed in ISDN-loaded polymeric systems—comprising an initial rapid release followed by sustained drug diffusion—is advantageous for wound healing, ensuring both immediate therapeutic action and prolonged drug availability [51]. Future studies should employ advanced release modeling, such as the Korsmeyer–Peppas equation, to further elucidate the governing mechanisms. In the context of controlled drug delivery, the release kinetics of ISDN from biopolymeric systems have been extensively studied. These systems often display a biphasic release pattern: an initial rapid release, followed by a sustained release phase. Such profiles are advantageous in wound healing, providing an immediate therapeutic effect while maintaining prolonged drug availability [52]. The release mechanism is frequently analyzed using models like the Korsmeyer–Peppas equation, which helps elucidate whether the drug release is governed by diffusion, polymer relaxation, or a combination of both [53].

The hemocompatibility study was conducted to obtain preliminary information on the biocompatibility of the systems. The hemocompatibility evaluation revealed that all SEI formulations induced hemolysis levels below 1.0%, well within the accepted safety threshold for biomaterials (<2%) [54]. Similarly, the control membrane (S) also exhibited hemolysis levels under 1.0%, indicating that it, too, meets the criteria for hemocompatibility. This result reinforces the suitability of both the SEI systems and the control membrane for applications involving direct contact with blood or vascularized tissues. Due to the fact that starch (S) is the major component in all SEI formulations, it was expected that both the SEI systems and the control would exhibit similar hemolysis levels. The comparable results observed confirm that the presence of additional components, such as ECM and ISDN, does not induce hemolytic activity, further corroborating that the SEI systems are non-hemolytic and safe for biomedical use. This finding underscores their suitability for biomedical applications involving direct blood contact. Collectively, the results suggest that SEI biopolymeric systems, particularly SEI3, present a promising platform for controlled drug delivery. Further research should focus on *in vitro* cell viability, *in vivo* assessments, long-term degradation behavior, and formulation refinements to optimize clinical applicability. In this context, although antioxidant and anti-inflammatory analyses were not included in the present work, they represent critical parameters for evaluating the bioactivity of materials intended for tissue repair. These assessments will therefore also be integral to future research, providing a more comprehensive understanding of the systems’ therapeutic potential.

## 5. Conclusions

This study introduces an innovative scaffold with improved mechanical stability, controlled degradation, moisture retention, and hemocompatibility, making it a promising alternative to conventional wound healing materials. The unique integration of S, dECM, and ISDN is designed to synergistically enhance the healing process, potentially offering superior performance over currently available biomaterials. The primary objective of this research was to synthesize and characterize this new biopolymeric system and evaluate its suitability for dermal wound healing applications.

The developed formulations exhibited favorable physicochemical characteristics, thermal stability, and excellent hemocompatibility, with hemolysis levels well below the accepted safety threshold (<2%). These attributes confirm their potential for biomedical applications, particularly those involving direct blood contact. Among the tested formulations, SEI3 demonstrated the most balanced mechanical properties, offering an optimal combination of strength and controlled flexibility. This makes it a strong candidate for applications requiring both structural integrity and durability. Additionally, SEI3 and SEI4 exhibited a more controlled isosorbide dinitrate (ISDN) release profile, effectively minimizing the initial burst effect while ensuring sustained drug diffusion. This feature is crucial for enhancing therapeutic efficacy by maintaining consistent drug levels over an extended period. The biphasic release behavior observed in these formulations, accurately modeled by the Korsmeyer–Peppas equation (R^2^ > 0.98), indicates a non-Fickian transport mechanism driven by both diffusion and polymer relaxation. Such a controlled release pattern is vital for optimizing drug bioavailability and mitigating potential side effects, thereby improving patient outcomes. Furthermore, the hemocompatibility results provide valuable insight into the biocompatibility of the developed systems, suggesting their potential safety for biomedical use. To advance the clinical applicability of the developed biopolymeric systems for wound healing, future studies should prioritize in vivo evaluations, long-term stability assessments, and formulation refinements that optimize therapeutic performance.

## Figures and Tables

**Table 1:** Formulation of the four biopolymeric systems studied as well as the control system.

Biopolymeric System	Starch (g)	Water (mL)	Glycerol (g)	dECM (g)	ISDN (mg)	Citric Acid (mg)
SEI1	2.5	50	0.75	0.175	20	25
SEI2	2.5	50	0.75	0.3125	20	25
SEI3	2.5	50	0.75	0.175	40	25
SEI4	2.5	50	0.75	0.3125	40	25
S	2.5	50	0.75	0	0	25

**Table 2:** Mechanical properties of biopolymeric systems submitted to tensile test.

Biopolymeric System	Strength (N)	Stress (N/mm^2^)	Strain (%)	Young’s Modulus (MPa)
SEI1	3.63 ± 0.10	0.36 ± 0.01	13.59 ± 0.79	2.68 ± 0.19
SEI2	2.50 ± 0.19	0.25 ± 0.01	19.64 ± 0.89	1.27 ± 0.05
SEI3	4.16 ± 0.29	0.41 ± 0.02	15.52 ± 0.71	2.68 ± 0.18
SEI4	2.30 ± 0.16	0.23 ± 0.01	14.83 ± 0.15	1.55 ± 0.10
S	4.08 ± 0.15	0.45 ± 0.01	12.45 ± 0.44	3.62 ± 0.11

**Figure A1:**
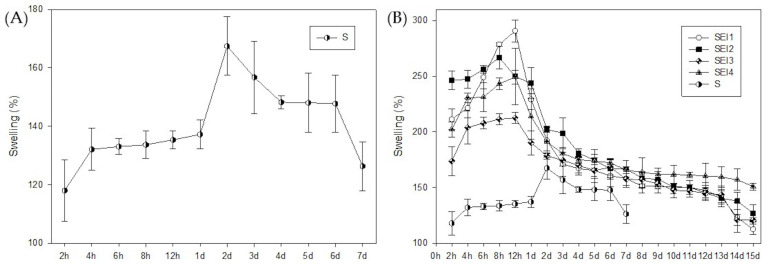
Swelling behavior of the systems over time. (**A**) Swelling percentage of the control membrane (S) measured over 7 days, showing a gradual increase in fluid uptake, with a peak at day 2. (**B**) Comparative swelling profiles of biopolymeric membranes (SEI1–SEI4) and control membrane (S) over a 15-day period. All SEI systems exhibited significantly higher swelling percentages than membrane S throughout the evaluation period.

## Data Availability

Data is contained within the article.

## Abbreviations

The following abbreviations are used in this manuscript:

ECM	Extracellular matrix
dECM	Decellularized extracellular matrix
S	Cassava starch
ISDN	Isosorbide dinitrate
SEI1	Starch/dECM7% (*w*/*w*)/ISDN20 mg biopolymeric system
SEI2	Starch/dECM12.5% (*w*/*w*)/ISDN20 mg biopolymeric system
SEI3	Starch/dECM7% (*w*/*w*)/ISDN40 mg biopolymeric system
SEI4	Starch/dECM12.5% (*w*/*w*)/ISDN40 mg biopolymeric system
ASTM	American Society for Testing and Materials
WVTR	Water vapor transmission rate
UTM	Universal testing machine
PBS	Phosphate-buffered saline
SBF	Simulated body fluid
FTIR	Fourier transform infrared spectroscopy
TGA	Thermogravimetric analysis
SEM	Scanning electron microscopy
EDTA	Ethylenediaminetetraacetic acid
RBCs	Red blood cells
ANOVA	Analysis of variance

## Appendix A

### Physicochemical Characterization of Cassava Starch

This appendix presents the physicochemical characterization of the cassava starch used in this study (Table A1). The analysis included iodine colorimetry to confirm starch presence and provide a qualitative indication of the amylose/amylopectin ratio based on coloration. Additionally, moisture content and ash content tests were conducted to evaluate the starch’s water retention capacity and the presence of inorganic residues, respectively.

**Table A1:** Physicochemical characterization of cassava starch.

Parameter	Methodology	Result	Interpretation
Iodine Colorimetry	Visual evaluation after iodine test	Blue–violet coloration	Confirms starch presence; coloration suggests intermediate amylose/amylopectin ratio
Moisture Content (%)	Gravimetric method at 105 °C	9.72 ± 0.09%	Within expected range for dried starch; indicates low water content
Ash Content (%)	Muffle furnace at 550 °C	0.25 ± 0.01%	Low inorganic residue, indicating good purity level
pH	pH meter in aqueous starch solution (1% *w*/*v*)	6.3 ± 0.2	Slightly acidic/neutral; within typical range for native cassava starch

## Appendix B

### Swelling Behavior of Starch-Only Control Membrane

Appendix B presents the swelling behavior of the control membrane (S) (Figure A1), shown in panel A, alongside the comparative swelling profiles of the SEI biopolymeric systems (SEI1–SEI4) in panel B. In panel A, the swelling percentage of membrane S is plotted over a 7-day period, demonstrating a gradual increase in fluid uptake during the first days, peaking around day 2 (~170%), followed by a slight decline in swelling capacity towards day 7. Panel B allows for direct comparison between the SEI formulations and the control membrane (S) over a 15-day period, highlighting the consistently lower swelling percentage of membrane S across all time points. Notably, by day 7, membrane S reached a swelling level comparable to that of the SEI systems at day 30, supporting the discussion that attributes this behavior to its smooth, non-porous morphology.
